# Characterizing neurological status in individuals with tetraplegia using transcutaneous spinal stimulation

**DOI:** 10.1038/s41598-023-48811-0

**Published:** 2023-12-06

**Authors:** Jeonghoon Oh, Michelle S. Scheffler, Catherine A. Martin, Jenny Dinh, Jony Sheynin, Alexander G. Steele, Dimitry G. Sayenko

**Affiliations:** 1 Department of Neurosurgery, Center for Translational Neural Prosthetics and Interfaces, Center for Neuroregeneration, Houston Methodist Research Institute, Houston, TX 77030 USA; 2 Department of Psychiatry and Behavioral Science, Texas A&M University Health Science Center, Houston, TX USA

**Keywords:** Neuroscience, Physiology, Medical research

## Abstract

Transcutaneous spinal stimulation (TSS) is emerging as a valuable tool for electrophysiological and clinical assessment. This study had the objective of examining the recruitment patterns of upper limb (UL) motor pools through the delivery of TSS above and below a spinal lesion. It also aimed to explore the connection between the recruitment pattern of UL motor pools and the neurological and functional status following spinal cord injury (SCI). In eight participants with tetraplegia due to cervical SCI, TSS was delivered to the cervical spinal cord between the spinous processes of C3–C4 and C7–T1 vertebrae, and spinally evoked motor potentials in UL muscles were characterized. We found that responses observed in UL muscles innervated by motor pools below the level of injury demonstrated relatively reduced sensitivity to TSS compared to those above the lesion, were asymmetrical in the majority of muscles, and were dependent on the level, extent, and side of SCI. Overall, our findings indicate that electrophysiological data acquired through TSS can offer insights into the extent of UL functional asymmetry, disruptions in neural pathways, and changes in motor control following SCI. This study suggests that such electrophysiological data can supplement clinical and functional assessment and provide further insight regarding residual motor function in individuals with SCI.

## Introduction

Approximately 18,000 spinal cord injury (SCI) incidents occur in the United States per year^1^, resulting in significant impairments of motor, sensory, and autonomic functions. Additionally, the financial burden associated with SCI is substantial^1^. A cervical SCI is often accompanied by a severe loss of function in the upper limbs (UL), particularly the hands^2, 3^. Even mild deficits in hand function and dexterity after cervical SCI lead to dramatic degradation of quality of life^4^. Regaining UL function represents the highest priority for individuals with cervical SCI^5^. Thus, the use of appropriate assessment measures is crucial to ensure effectiveness of rehabilitation strategies, identify individuals who respond well to treatment, and monitor rehabilitation outcomes following SCI. Two population-specific assessment measures are frequently used in SCI research for both descriptive and evaluative purposes. A *clinical* assessment entitled the International Standards for Neurological Classification of Spinal Cord Injury (ISNCSCI)^6^ systematically examines the motor and sensory functions post-SCI. Based on key motor and sensory levels, the ISNCSCI provides discriminative information on both type (complete or incomplete) and level of injury. In particular, the UL motor score (ULMS) component examines residual bilateral motor function post injury. The Graded Redefined Assessment of Strength, Sensibility, and Prehension (GRASSP)^7^ is a *functional* assessment that includes UL strength, sensory, and grasp subtests, and quantifies UL function post SCI. A combination of ISNCSCI-ULMS and GRASSP has been shown to be the best predictors of UL function^8^. However, a major limitation of these clinical and functional tools is the inability to characterize the status of spinal motoneurons and network below the lesion. A better understanding of the spinal network status and function is critical for several factors, including the response to descending commands, employment of the spinal reflex circuitry, and its ability to respond to afferent changes. Thus, the relationship between the state of the spinal network and neurological and functional status after SCI is likely not only to contribute to the characterization and prediction of recovery for UL motor function, but also to tailor rehabilitation therapy of affected individuals.

Electrical stimulation of the spinal cord is an emerging tool in clinical practice for facilitating the recovery of sensory and motor function in individuals with SCI. In addition, spinal stimulation is a valuable method for electrophysiological characterization of residual motor function after SCI^9–11^. Transcutaneous spinal stimulation (TSS) is a non-invasive method that can produce motor responses via Ia afferents—α-motoneuron synapses, but also is capable to activate other neural components within the spinal cord, including interneurons, ascending sensory fibers in the dorsal columns, descending motor tracts, and other polysynaptic pathways^12–16^. The ability of TSS to evoke and modulate responses of spinal circuitries was well demonstrated in lower limb (LL) studies, making it a promising approach for the treatment and evaluation of SCI^17–19^. Recent studies in neurologically intact individuals demonstrated that TSS delivered over the cervical spinal cord resulted in a preferential activation of proximal and distal UL muscles depending on the site of stimulation^20, 21^. Despite these findings, it remains uncertain whether TSS can be used as an appropriate assessment method to detect sensorimotor changes after cervical SCI caused by disrupted communication between the brain and spinal cord, altered sensory input transmission through the spinal cord, directly affected motor pools, and other changes in the spinal cord circuitry^22, 23^. It may be beneficial, therefore, to utilize TSS to better understand the effects of SCI on the response of motor pools in individuals with cervical SCI, given the complex nature of this condition. This study’s objectives were to employ TSS of the cervical spinal cord to characterize the response of motoneurons to stimulation and quantify their viability after SCI. We hypothesized that the pattern of recruitment of motor pools projecting to UL muscles recorded by electrophysiological assessment will correspond to neurological or functional status of individuals with SCI as determined by ISNCSCI and GRASSP strength scores.

## Results

### Magnetic resonance imaging

**Figure 1:**
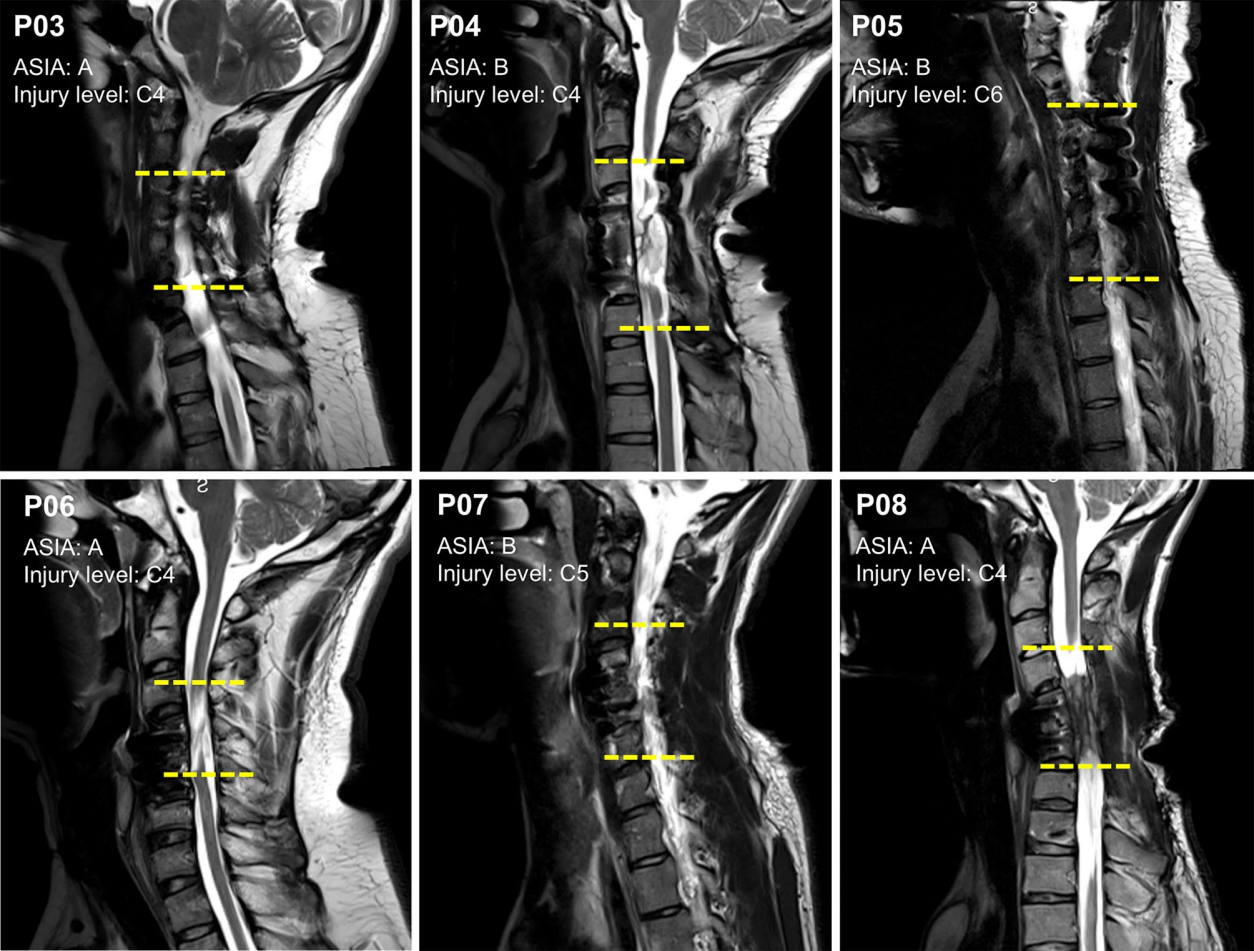
Structural magnetic resonance imaging (MRI) scans depicting sagittal sections within the cervical region. Sagittal slices from six participants with cervical spinal cord injury classified as AIS A and B. The yellow horizontal lines indicate the location of the cervical lesion between the upper and lower vertebrae.

Figure 1 displays sagittal slices obtained from the cervical level of six participants with SCI classified as AIS A and B to verify the level of injury between C4 and C7 vertebrae. Neuroimaging data for two participants (P01 and P02), both with AIS C, was not available. The yellow horizontal lines indicate the location of the cervical lesion between the upper and lower vertebrae.

### Functional assessment

Overall, there were individual differences in ISNCSCI UL motor and GRASSP strength scores across the key proximal (upper arm and forearm including DEL, BIC, ECR, and TRIC) and distal (hand including ED, FDP, OP, FPL, ADM, and FDI) muscles for each UL (Supplementary Table S1). Specifically, all eight participants demonstrated at least a 2/5 GRASSP strength score for DEL and BIC which are innervated by C5–C7. In contrast, muscles innervated by C8–T1 consistently exhibited 0/5 or 1/5 strength scores across both ULs in each participant. Functionally, this translated to individuals with SCI being unable to complete tasks requiring the use of key distal muscle groups. The distal deficits were more evident particularly in those with severe injuries (AIS A and B). While ISNCSCI examines motor scores of fewer muscle groups, the pattern of proximal preservation and distal paralysis was also evident with use of this tool to measure UL impairment. We observed differences between left and right muscle strength scores in GRASSP (*p* < 0.01), whereas no significant differences were found in ISNCSCI ULMS (*p* = 0.089). In order to assess disparities between proximal and distal muscles, we categorized the spines into C5–C7 and C8–T1 for ISNCSCI based on Table 2, and the muscles into DEL-TRIC and ED-FDI for GRASSP. This revealed significant distinctions between proximal and distal muscles for both ISNCSCI ULMS (*p* < 0.001) and GRASSP strength scores (*p* < 0.001).

For each participant, ISNCSCI ULMS are shown in Supplementary Table S1. At the C5 level, all participants showed active movement with full range of motion against gravity for both ULs. P01 and P02 demonstrated higher ISNCSCI ULMS for the left UL below C8, while the right limb scored a “0” below C8. This corresponded with the preferred/dominant hand use post injury. However, P01, P02, and P05 demonstrated residual function in muscles innervated by C8–T1 in their left hands, as shown by several non-zero scores. Generally, P02 and P05 demonstrated higher proximal UL segmental motor scores than any other participants. For example, P02 and P05 were able to perform full range of motion against gravity for both ULs in C5–C6 distributions. However, P03, P04, P06, P07, and P08 exhibited no distal UL segmental motor scores for both ULs in C8–T1 distributions. Overall, all participants showed higher scores for rostral than caudal cervical spinal areas, which is consistent with a cervical SCI clinical presentation. Based on their GRASSP strength scores in Supplementary Table S1, all participants demonstrated higher strength scores for proximal muscles than distal muscles. There was, however, a large discrepancy among strength in distal muscles for all participants. In addition, P01, P04, P05, P06, and P07 demonstrated a higher strength score for their left-hand muscles, while P03 and P08 showed higher strength score for the right-hand muscles. P02 demonstrated equal total strength scores bilaterally.

**Figure 2:**
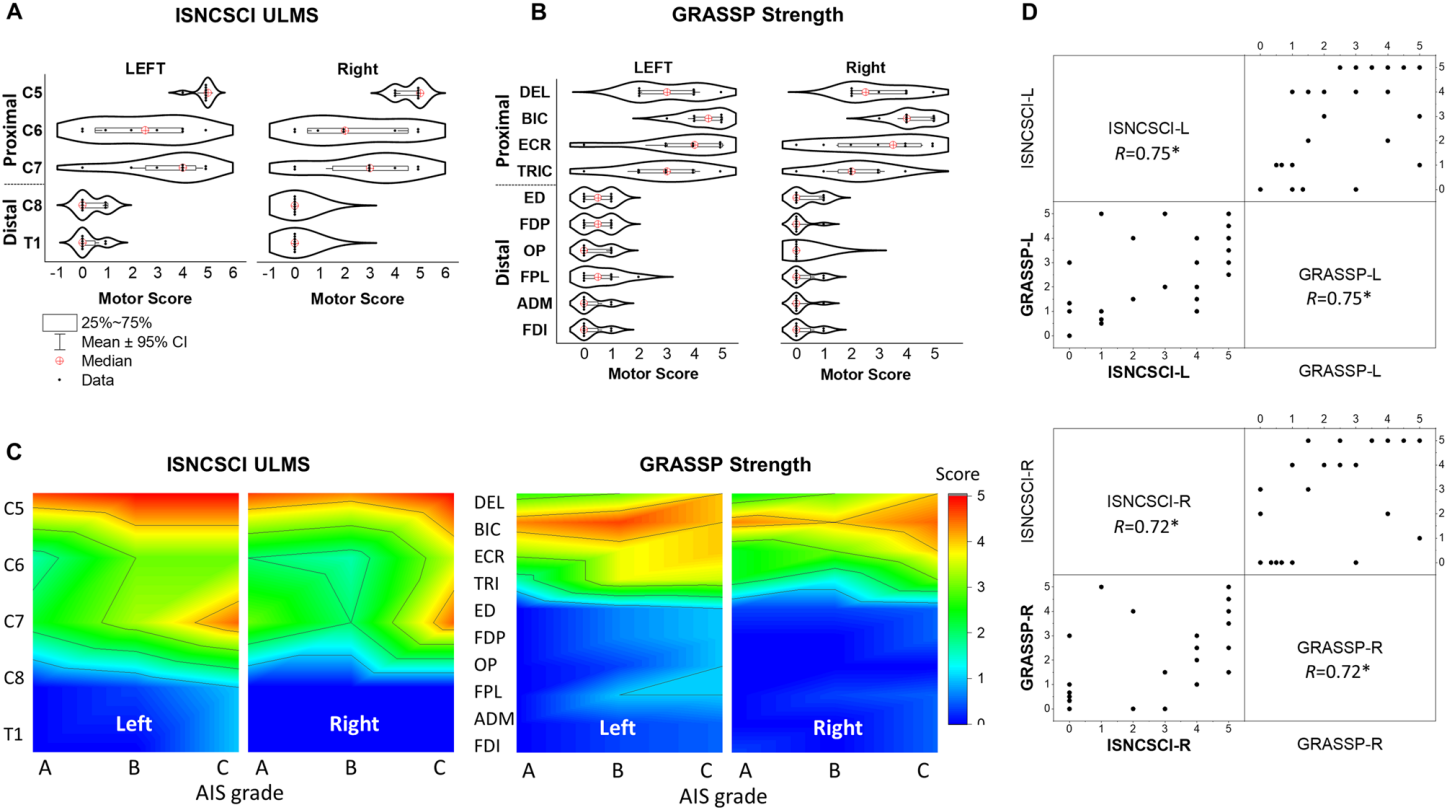
Functional assessment scores and correlation coefficient between the assessments. (**A**) Violin plots of the ISNCSCI upper-limb motor scores (ULMS) and, (**B**) GRASSP strength scores for all participants with spinal cord injury for both the left and right limbs**.** (**C**) Heatmap of the distribution of ISNCSCI ULMS and GRASSP strength scores, considering the categorized grades of the American Spinal Injury Association Impairment Scale (AIS) A, B, and C for both the left and right limbs. (**D**) Scatter matrix plot with Pearson’s correlation coefficient between ISNCSCI ULMS and GRASSP strength scores for both the left and right limb. *ISNCSCI* International Standards for Neurological Classification of Spinal Cord Injury, *AIS* American Spinal Injury Association Impairment Scale, *GRASSP* Graded Redefined Assessment of Strength, Sensibility, and Prehension, version 1, *DEL* anterior deltoid, *BIC* biceps brachii, *ECR* extensor carpi radialis, *TRIC* triceps brachii, *ED* extensor digitorum, *FDP* flexor digitorum profundus, *OP* opponens pollicis, *FPL* flexor pollicis longus, *ADM* abductor digiti minimi, *FDI* first dorsal interosseous. ISNCSCI: 0 = total paralysis, 1 = palpable or visible contraction, 2 = active movement, full range of motion (ROM) with gravity eliminated, 3 = active movement, full ROM against gravity, 4 = active movement, full ROM against gravity and moderate resistance in a muscle specific position, 5 = (normal) active movement, full ROM against gravity and full resistance in a muscle specific position expected from an otherwise unimpaired person. GRASSP: 0 = No palpable or visible muscle contraction, 1 = Palpable or visible muscle contraction, 2 = Moves full ROM with gravity eliminated, 3 = Moves full ROM against gravity without added resistance, 4 = Holds position of resistance against moderate resistance, 5 = Holds position of resistance against maximal resistance.

Figure 2 shows averaged ISNCSCI UL motor (Fig. 2A) and GRASSP strength scores (Fig. 2B) for all participants. The ISNCSCI UL motor scores at C5–C7 levels for both left and right sides correspond with the GRASSP strength scores. Based on the GRASSP strength scores, the proximal muscles (DEL, BIC, TRIC, and ECR) were stronger when compared to the distal UL muscles (ED, OP, FPL, FF, ADM, and FDI). Figure 2C illustrates the heatmap of ISNCSCI UL motor and GRASSP strength scores for both ULs for all participants after considering AIS grade (A, B, and C). Participants with AIS C, at the C7 level of the cervical spine showed higher motor scores in ISNCSCI ULMS compared to those classified as AIS A and B. The ISNCSCI UL motor and the GRASSP strength scores had a strong correlation for both the left and right limbs (Left: *r* = 0.75, Right: *r* = 0.72, *p* < 0.05) (Fig. 2D).

### Muscle recruitment of upper-limb motor pools

#### Muscle recruitment curves during TSS above and below lesion

**Figure 3:**
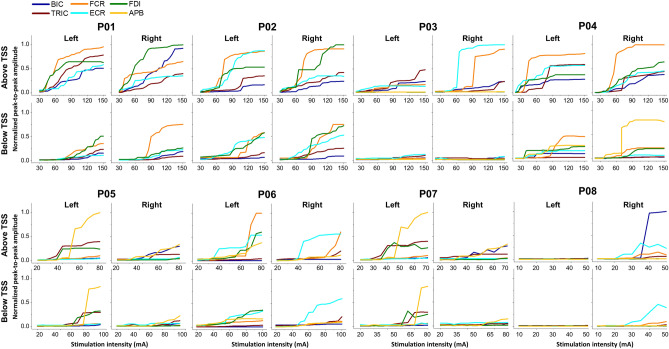
Spinally evoked motor recruitment curves in eight participants with cervical spinal cord injury during TSS above and below the lesion for left and right limbs for all participants. Both left and right upper-limb muscle responses elicited during cervical TSS with an electrode placed between the spinous process of C3–C4 (above lesion) and C7–T1 (below lesion) vertebrae are shown for all participants. The intensity of stimulation was adjusted based on the maximum tolerated intensity of the participant, which ranged from 10 to 150 mA. *AIS* American Spinal Cord Injury Association Impairment Scale, *TSS* transcutaneous spinal stimulation, *BIC* biceps brachii, *TRIC*: triceps brachii, *FCR* flexor carpi radialis, *ECR* extensor carpi radialis, *FDI* first dorsal interosseous, *APB* abductor pollicis brevis muscles.

Figure 3 presents the recruitment curves of all tested UL muscles during TSS above and below the lesion for all ULs of all participants. Overall, the responses were higher when TSS was delivered above the lesion, except for hand muscles in the case of P03 and P08, who did not exhibit any response in hand muscles during TSS either above or below the lesion. This is consistent with the ISNCSCI ULMS and GRASSP strength scores of P03 and P08 (Supplementary Table S1).

**Figure 4:**
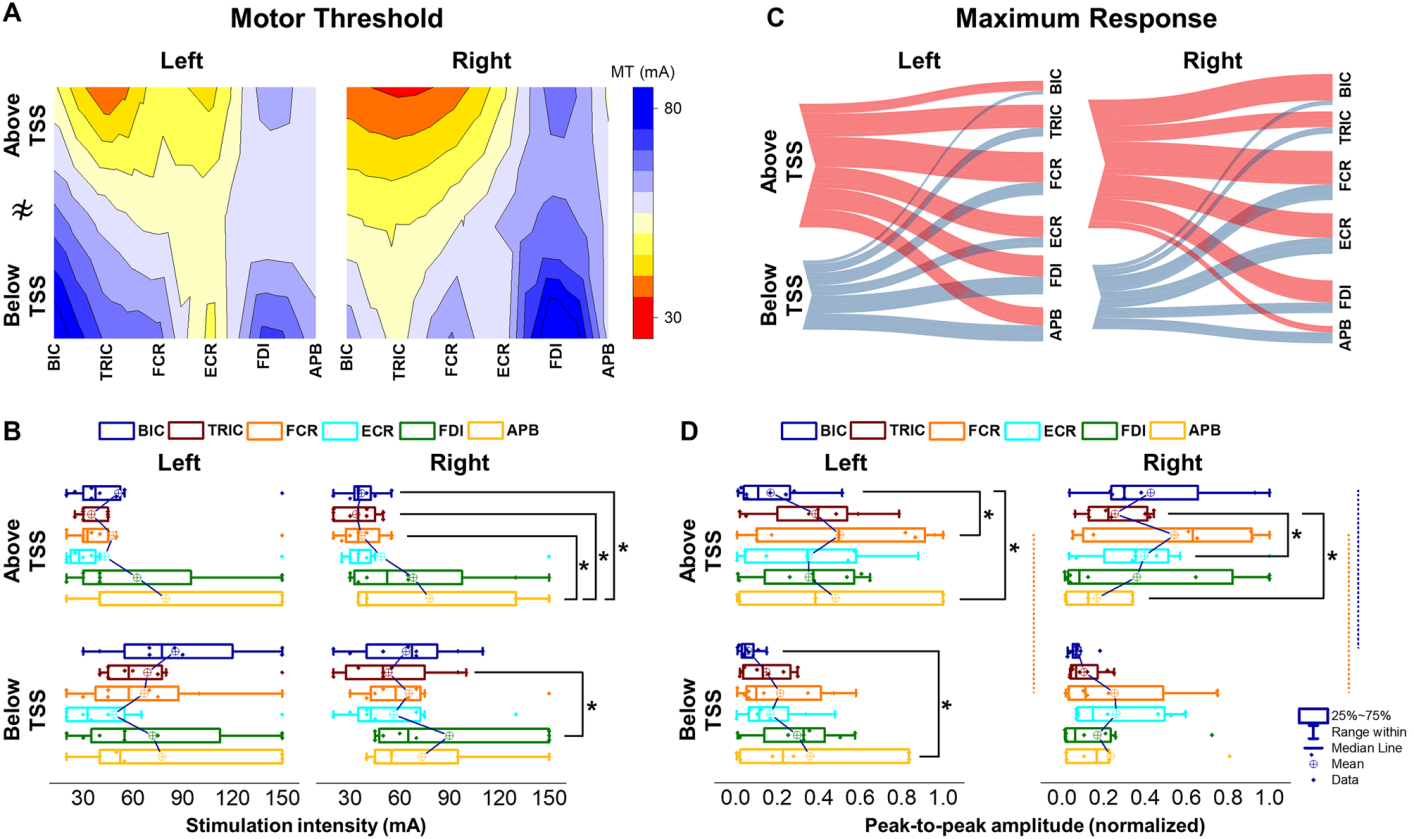
Comparison of muscle activations elicited above (C3–C4 vertebrae) and below the lesion (C7–T1 vertebrae) of cervical TSS. (**A**) Heatmap of motor threshold intensity for all tested UL muscles during TSS above and below the lesion for all participants. (**B**) Average of the motor threshold intensity during TSS above and below the lesion for both limbs. (**C**) Parallel coordinates plots were generated by averaging the maximum response values across all participants, depicting the relationship between TSS above and below the lesion to the UL muscles. Thickness of connections denotes the amplitude of the maximum motor response for both limbs. (**D**) Average of the maximum response during TSS above and below the lesion for both limbs. Box range was set as percentage 25–75%, bold vertical red lines in boxplots present the median values, and whiskers indicate the 95% confidence interval. A post-hoc Holm–Bonferroni correction was carried out for multiple comparison within figures. Significant differences are indicated with vertical lines. Dotted lines: *p* < 0.05; dashed lines: *p* < 0.01; and solid lines: *p* < 0.001. *TSS* transcutaneous spinal stimulation, *BIC* biceps brachii, *TRIC* triceps brachii, *FCR* flexor carpi radialis, *ECR* extensor carpi radialis, *FDI* first dorsal interosseous, *APB* abductor pollicis brevis muscles.

Figure 4A shows the heatmap of the motor threshold (MT) in both the left and right UL muscles during TSS above and below the lesion. There were no differences in MT for left limb when TSS was delivered both above and below the lesion while differences in MT were found between the right UL muscles during TSS both the above and below the lesion (Left: X^2^(5) = 7.257, *p* = 0.20; Right: X^2^(5) = 9.465, *p* < 0.05) (Fig. 4B). When TSS was delivered above the lesion, the MT of the right BIC, TRIC, and FCR exhibited a lower MT than APB (*p* < 0.05). During TSS below the lesion, the MT of the right TRIC showed a lower MT than FDI (*p* < 0.05).

Figure 4C illustrates the relationship between TSS above and below the lesion to the degree of activation for tested UL muscles for the left and right limbs for all participants. When TSS was delivered above the lesion, larger muscle responses were evoked for all tested UL muscles compared to TSS below the lesion. There were differences in MaxR between TSS above and below the lesion for both the left and right limbs (Left: X^2^(5) = 6.342, *p* < 0.05, Right: X^2^(5) = 5.209, *p* < 0.05). Post-hoc comparisons revealed that MaxR was higher for the right BIC and both FCR during TSS above the lesion compared to below the lesion (*p* < 0.05, Fig. 4D). When TSS was delivered above the lesion, the MaxR of the left APB was higher than the left BIC and FCR (*p* < 0.05) while the MaxR of the right TRIC was higher than the right ECR and APB (*p* < 0.05).

#### Bilateral muscle recruitment of upper-limb motor pools during cervical TSS

**Figure 5:**
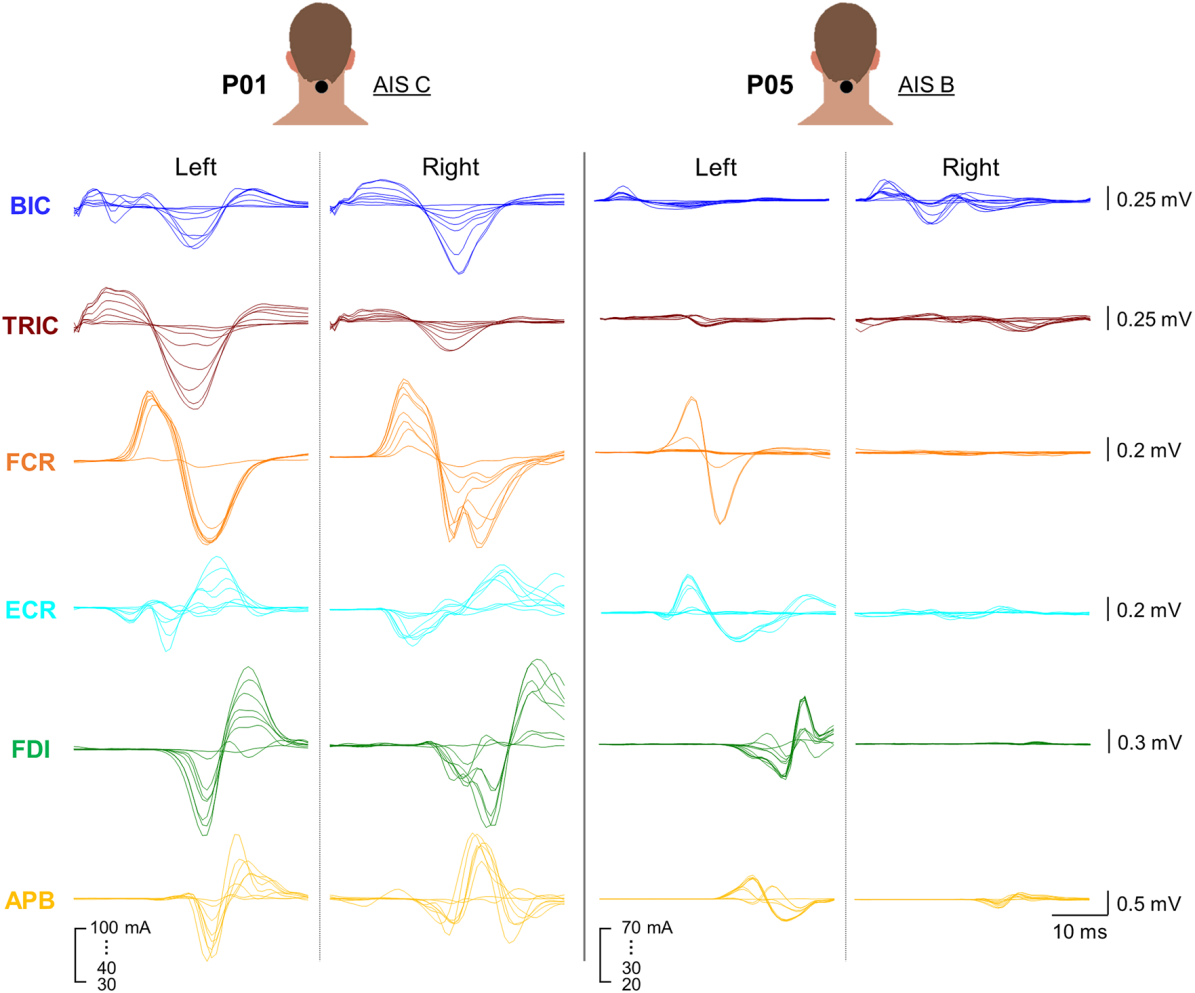
Exemplary bilateral amplitude of upper-limb muscle responses to cervical TSS. Both left and right upper-limb muscle responses elicited with the stimulation with an electrode placed between the spinous process of C3–C4 vertebrae are shown for a representative participant classified as AIS C (P01) and AIS B (P05). Stimulation intensity ranged from 30 to 100 mA for P01, and 20 to 70 mA for P05. *TSS* transcutaneous spinal stimulation, *BIC* biceps brachii, *TRIC* triceps brachii, *FCR* flexor carpi radialis, *ECR* extensor carpi radialis, *FDI* first dorsal interosseous, *APB* abductor pollicis brevis muscles.

Figure 5 demonstrates the waveforms of asymmetric evoked responses in all tested muscles between the left and right UL during TSS above the level of injury in a representative participant who classified as AIS C (P01) and AIC B (P05). During TSS above the lesion for P01 (AIS C), the response observed in the left TRIC was greater than the right TRIC while other UL muscles exhibited nearly equal levels of response between the limbs. This finding is consistent with the asymmetry observed between the limbs in the GRASSP strength scores for TRIC (left = 3, right = 2) (Supplementary Table S1). However, the ISNCSCI ULMS for TRIC showed equal scores for both the left and right sides, with both scoring 4. Although spinally evoked motor potentials innervated by C3–C4 (above the lesion) were observed in both UL of P01, the ISNCSCI ULMS and GRASSP strength scores still showed several instances of zero scores for each UL (Supplementary Table S1). During TSS above the lesion for P05 (AIS B), a notable asymmetry was observed in all tested UL muscles except TRIC, with a higher response in the left limb compared to the right limb.

**Figure 6:**
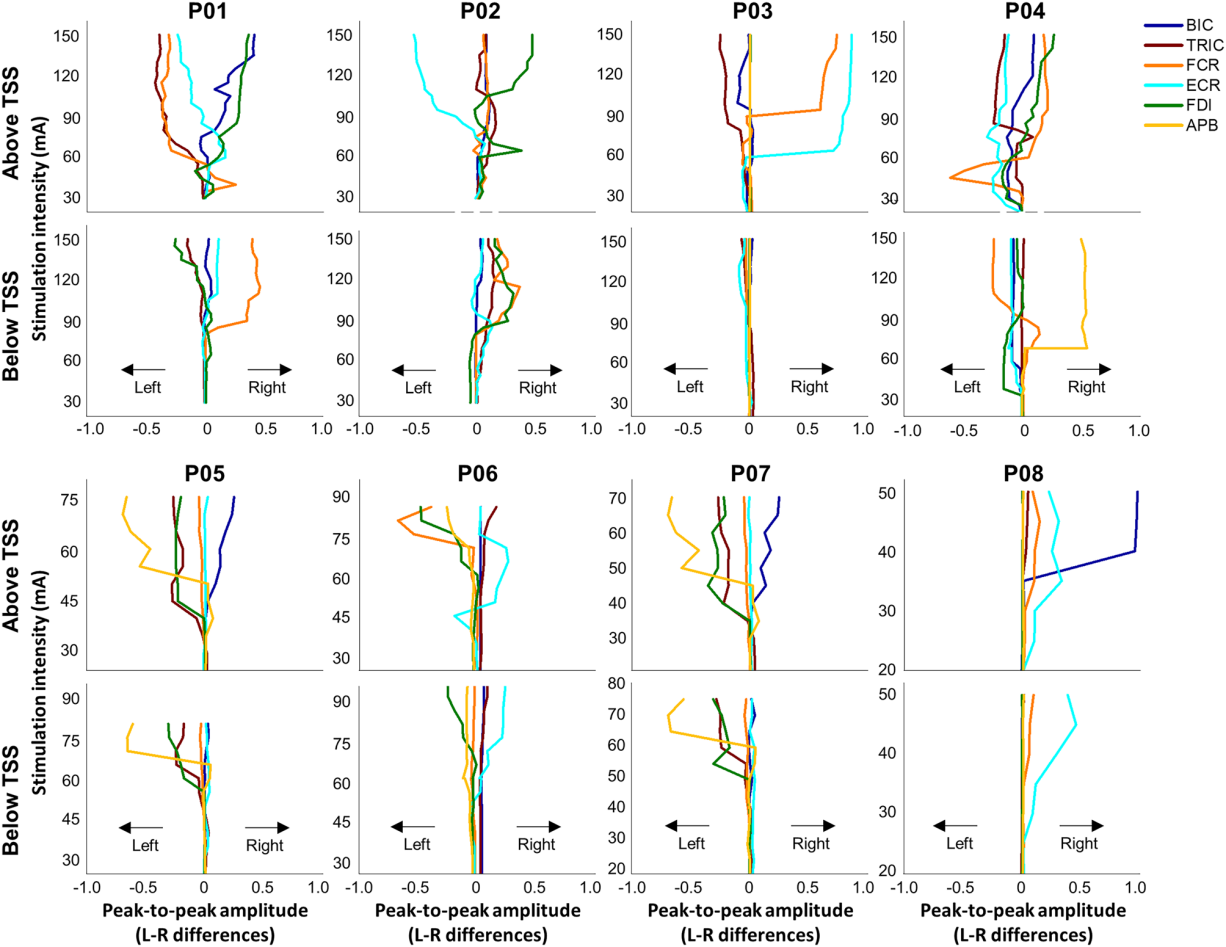
Limb asymmetric muscle response during TSS above and below the lesion in participants with cervical spinal cord injury. The limb amplitude differences between the limbs for all tested UL muscles during stimulation above (C3–C4 vertebrae) and below (C7–T1 vertebrae) the lesion for all participants. The intensity of stimulation was adjusted based on the maximum tolerated intensity of the participant, which ranged from 10 to 150 mA. The line plots shifted to the left indicate that the left limb had a greater amplitude than the right limb, whereas the line plots shifted to the right indicate that the right limb had a greater amplitude than the left limb. *TSS* transcutaneous spinal stimulation, *BIC* biceps brachii, *TRIC* triceps brachii, *FCR* flexor carpi radialis, *ECR* extensor carpi radialis, *FDI* first dorsal interosseous, *APB* abductor pollicis brevis muscles.

Differences in all tested UL muscles between the left and the right limbs during TSS above and below the lesion are shown in Fig. 6, for each participant. Specifically, P01 demonstrated a pronounced asymmetrical response in UL muscles during TSS above the lesion. When TSS was delivered above the lesion, the left TRIC, FCR and ECR muscles exhibited a greater response than the right limb, while the right BIC and FDI muscles showed a greater response than the left limb. P02 exhibited relatively symmetrical responses for BIC, TRIC, FCR during TSS above the lesion. This corresponded to an equivalent total strength score in the UL muscles observed in the ISNCSCI ULMS and GRASSP strength scores (Supplementary Table S1). P03 exhibited a pronounced asymmetrical response in the forearm muscles (FCR and ECR) in the right UL during TSS above the lesion, whereas the upper arm muscles (BIC and TRIC) responded more in the left UL. However, P03 and P08 did not show any responses in hand muscles (FDI and APB) during TSS delivered both above and below the lesion. P04 showed the most pronounced APB response in the right UL during TSS delivered below the lesion, in contrast to the other participants. This finding contradicts P04’s residual function in the muscles of the right hand that are innervated by C8–T1, as evidenced by several non-zero scores in both the ISNCSCI ULMS and GRASSP strength scores (Supplementary Table S1). P05 exhibited a greater response in TRIC and hand muscles (FDI and APB) on the left side during TSS delivered both above and below the lesion compared to the right UL. This is noteworthy because P05 demonstrated higher residual function in the left limb, as indicated by the ISNCSCI ULMS and GRASSP strength scores in Supplementary Table S1. P06 showed a greater response in FCR and hand muscles (FDI and APB) on the left side compared to the right side during TSS delivered above the lesion. P07 exhibited a greater response in TRIC and hand muscles (FDI and APB) on the left side during TSS delivered both above and below the lesion compared to the right UL while BIC response was higher during TSS delivered above the lesion on the right side compared to the left side. P08 did not show any responses in tested UL muscles on the left side while right BIC was higher during TSS delivered above the lesion compared to any other tested UL muscles.

**Figure 7:**
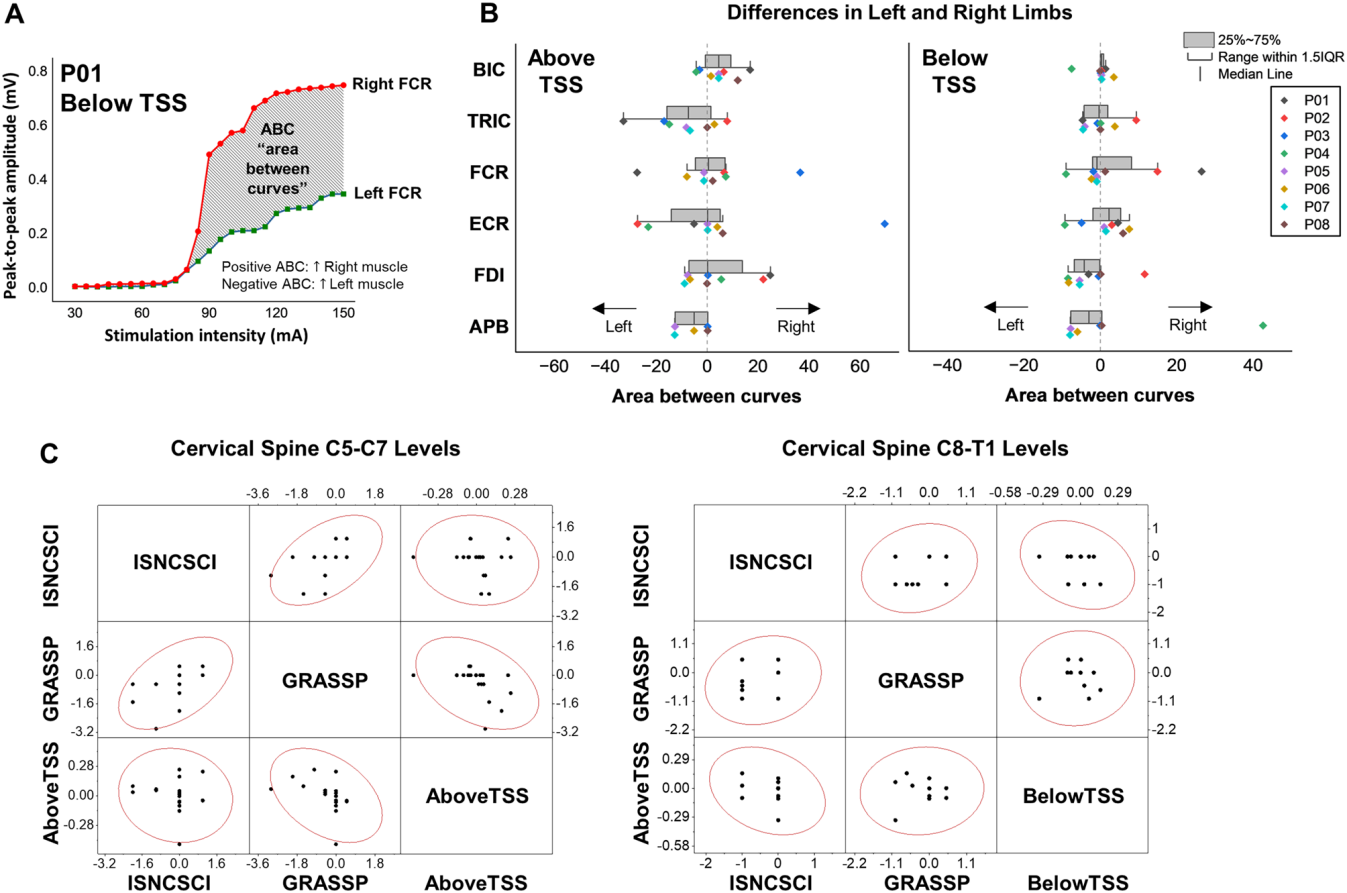
Relationship in bilateral limb asymmetric between clinical/functional scores and spinally evoked motor potential. (**A**) Sample analysis of area between curves (ABC) between the right and left muscles for P01 during stimulation below the lesion. (**B**) ABC of individual data in upper-limb muscles during stimulation above and below the lesion. The dots shifted to the left (negative values) indicate that the left muscle had a greater amplitude than the right muscle, whereas the dots shifted to the right (positive values) indicate that the right muscle had a greater amplitude than the left muscle. (**C**) Scatter matrix plot with Pearson’s correlation coefficient in left and right limb asymmetry between ISNCSCI ULMS and GRASSP strength scores and motor evoked potentials in UL motor pools during TSS above the lesion (left) and below the lesion (right). *TSS* transcutaneous spinal stimulation, *BIC* biceps brachii, *TRIC* triceps brachii, *FCR* flexor carpi radialis, *ECR* extensor carpi radialis, *FDI* first dorsal interosseous, *APB* abductor pollicis brevis muscles.

Figure 7A illustrates an example of area between curves (ABC) calculation between the left and right muscles for P01 during stimulation below the lesion. A negative ABC value indicates greater amplitude in the left muscle, while a positive ABC value indicates greater amplitude in the right muscle. Figure 7B presents limb asymmetry, as determined by ABC calculations for all participants in UL muscles during stimulation both above and below the lesion. For P01, ABC analysis revealed a higher response in the left TRIC and FCR muscles compared to the right muscles during stimulation above the lesion. Conversely, P02 exhibited a stronger response in the right UL muscles compared to the left muscles during both stimulation sites, except for ECR during stimulation above the lesion. P03 displayed increased response in the right FCR and ECR muscles compared to the left muscles during stimulation above the lesion. P06 and P08 did not show pronounced asymmetry between their UL muscles.

Figure 7C illustrates the relationship between the bilateral limb asymmetry observed in functional assessments from the ISNCSCI ULMS and GRASSP strength scores, and the motor evoked potential in UL motor pools during TSS delivered both above and below the injury, across different levels of the cervical spine. There was a moderate correlation (*r* = 0.51, *p* < 0.05) between left and right limb ISNCSCI ULMS and GRASSP strength scores across the cervical spine C5–C7 levels. However, there was a weak correlation observed between the asymmetry of left and right limbs in the functional assessments and the asymmetry of muscle response during TSS for the cervical spine levels C5–C7 and C8–T1 (Fig. 7C).

#### Muscle recruitment curves during transcranial magnetic stimulation

**Figure 8:**
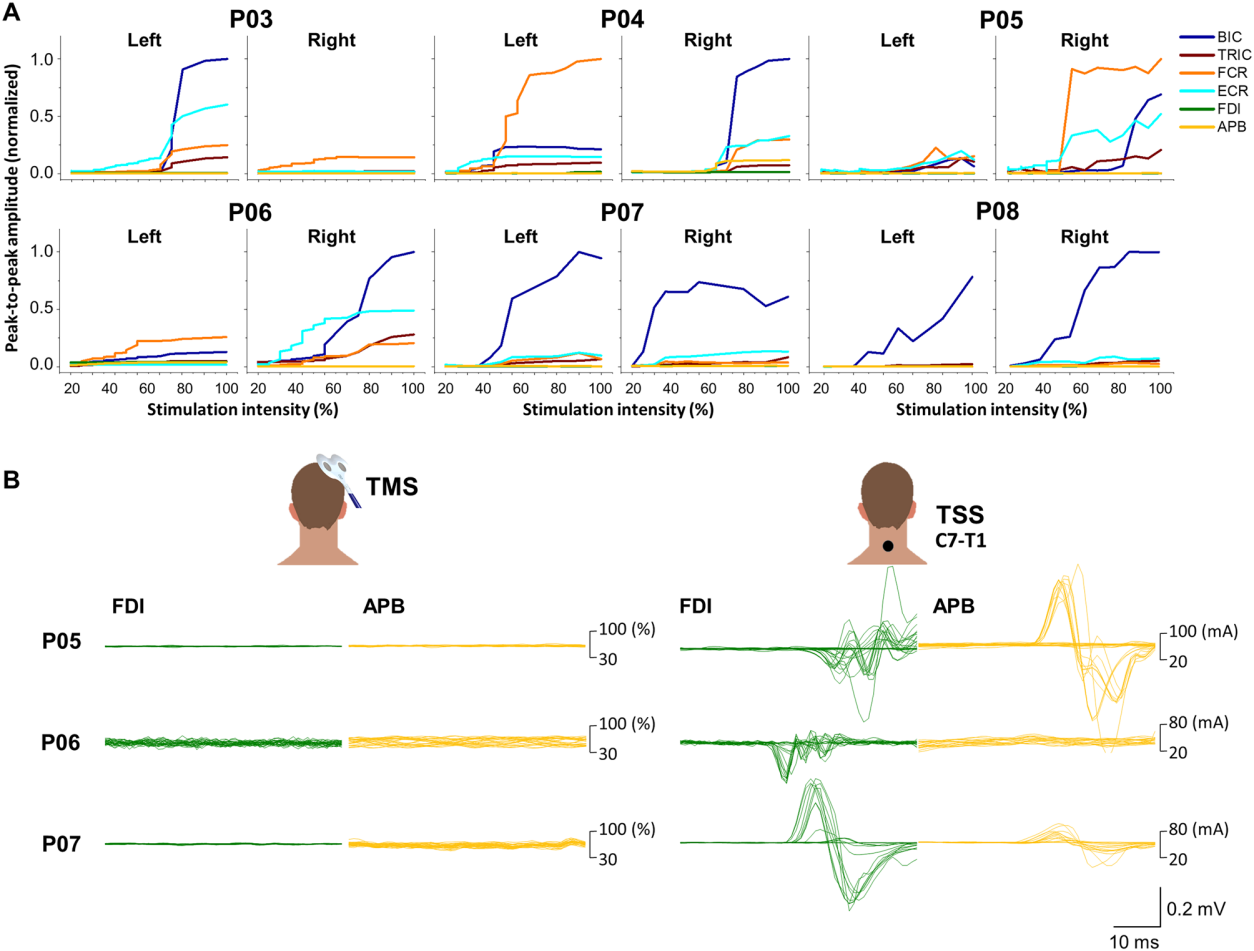
Motor recruitment curves obtained using transcranial magnetic stimulation (TMS) delivered over the left and right motor cortex in participants with cervical spinal cord injury who classified as AIS A and B. (**A**) Both left and right upper-limb muscle responses elicited with TMS over the left and right motor cortex are shown for all participants who classified as AIS A and B. (**B**) The evoked responses in left hand muscles (FDI and APB) during TMS of the left (P06) and right hemisphere (P05 and P07) and cervical TSS delivered at the C7–T1 vertebral level (i.e., below the lesion) in participants who showed the hand muscle responses during TSS. *TMS* transcranial magnetic stimulation, *TSS* transcutaneous spinal stimulation, *BIC* biceps brachii, *TRIC* triceps brachii, *FCR* flexor carpi radialis, *ECR* extensor carpi radialis, *FDI* first dorsal interosseous, *APB* abductor pollicis brevis muscles.

Figure 8A shows muscle recruitment curves of UL motor pools during transcranial magnetic stimulation (TMS) delivered over the left and right motor cortex for six participants with cervical SCI classified as AIS A and B. Overall, during TMS, all tested participants showed either minimal or no response in their hand muscles (FDI and APB). Notably, P07 and P08 only exhibited BIC response in both limbs during TMS. However, there were no observable responses in the rest of the tested UL muscles. In Fig. 8B, a comparison is made between the evoked responses in left hand muscles (FDI and APB) during TMS of the left (P06) and right hemisphere (P05 and P07) and cervical TSS delivered at the C7–T1 vertebral level (i.e., below the lesion) in participants who showed the hand muscle responses during TSS. Two participants classified as AIS B (P05 and P07) and one as AIS A (P06) displayed responses in their hand muscles during TSS, despite the absence of motor potentials evoked by TMS. Participants classified as AIS A (P03 and P08) did not exhibit any hand muscle responses during TSS. P04 demonstrated both TMS-evoked and TSS-evoked motor potentials.

## Discussion

We found that deficiencies in the distal muscles detected by clinical and functional assessments were evident, particularly in participants with severe injuries classified as AIS A and B. However, electrophysiological assessment using TSS delivered above and below the lesion in participants with cervical SCI can provide a more detailed characterization of UL asymmetry and help quantify the remaining motor function after SCI. Although the corticospinal pathway is severely impacted following SCI, we demonstrated the responses of motor pools projecting to proximal and distal UL muscles. While clinical and functional assessments may have limitations in measuring UL impairments after SCI, electrophysiological data can provide valuable insights into disruptions of neural pathways and changes of motor control.

### TSS delivered above and below lesion reveals different recruitment patterns of UL motor pools after cervical SCI

We explored the hypothesis that after cervical SCI, motor pools projecting to UL muscles will respond to TSS depending on their viability and functional status. Although our data showed that the spinal cord networks can be effectively engaged during stimulation along the rostrocaudal axis, the findings from participants with cervical SCI differ from previous findings obtained in neurologically intact subjects^20^. For example, participants with SCI demonstrated minimal UL muscle responses during TSS below the lesion, as evidenced by high MT and low maximum response (MaxR). This observation could be attributed to changes in the spinal circuitry following chronic SCI. As the injury duration progresses, motor pools might become under-stimulated, potentially leading to a loss of connections within both the multisegmental network and the descending projections. Consequentially, this could result in reduced synaptic efficiency and decreased excitability of motoneurons^16, 22–24^. Additionally, there may be alterations in the properties of interneurons, which can affect the reflex circuitry^25^. At the same time, preserved motor responses in hand muscles innervated by motor pools located below the lesion demonstrate the viability of these pools even after motor complete paralysis in most of tested participants with SCI.

### TSS allows assessment of bilateral recruitment of UL motor pools after cervical SCI

Symmetry in UL function is vital for everyday tasks in people with SCI. While several studies have focused on assessing asymmetry in lower limb muscles, there has been a notable shortage of research investigating asymmetry in the ULs. Some studies explored differences between the more affected and less affected sides following SCI utilizing UL robotic exoskeleton technology^26^ and functional measurements^27^. Cervical SCI in humans often results in bilateral anatomical damage and asymmetrical functional deficits in the hands and arms. According to electrophysiology and neuroimaging studies, the bilateral lesions exhibit asymmetry, whereby there is greater damage observed on one side of the spinal cord in comparison to the other side^28, 27^. Asymmetries in the distribution of dorsal root collateral sprouting after spinal cord lesions in animals^29^, as well as physiological^30–32^ and behavioral^31^ outcomes in humans with SCI, have also been reported. While improvement in UL function after SCI is an important goal in the rehabilitation process, clinical and functional assessment tools cannot always show significant differences in the magnitude of motor and functional recovery between the dominant and non-dominant limbs following cervical SCI^33^.

Electrophysiological data can provide valuable information about disturbances and sensorimotor changes post SCI, and can complement clinical assessment tools to guide the rehabilitation process. Specifically, we identified two strengths of electrophysiological guidance. First, we found that cervical TSS evoked potentials can be more sensitive in detecting asymmetry of UL muscles and spinal cord involvement post injury than clinical and functional assessments alone. For example, in our participants, ISNCSCI ULMS and GRASSP strength scores showed limited residual muscle function in distal muscles (innervated by C7–T1), and four participants demonstrated scores of zero for distal muscles bilaterally as measured by ISNCSCI ULMS. However, the recruitment of motor pools was more effective in demonstrating which UL distal muscles, including on right versus left sides, remained intact following injury. Second, participants with SCI showed asymmetrical recruitment of the motor pools during cervical TSS, which was poorly associated with the preservation of asymmetrical bilateral motor function observed during functional and clinical assessments. Asymmetrical recruitment of motor pools during TSS along the rostrocaudal axis, possibly due to variability in lesion size or area, indicate that spinally evoked potentials can be used in physiological, anatomic, and clinical studies to assess the degree of symmetry of the respective spinal reflex circuits.

### Corticospinal pathways in cervical SCI

The corticospinal tract is a major neural pathway between the cerebral cortex and the spinal cord, which plays a crucial role in mediating voluntary distal movements^34–36^. Following injury to the central nervous system, such as SCI, the corticospinal tract can undergo plasticity and reorganization, establishing new connections and compensating for lost function, thus contributing to functional recovery. Motor evoked potentials induced by TMS have been proposed as a potential biomarker of the severity of neurological deficits, but their correlation with function may not be consistent^37, 38^. Nevertheless, motor evoked potential can provide greater sensitivity than clinical examination alone in detecting corticospinal deficit. In the present study, the observed decline of motor evoked potentials induced by TMS was in alignment with the decline in motor function (Fig. 8A). Remarkably, cervical TSS retained its capacity to elicit responses in hand muscles in some participants (i.e., P05, P06, and P07) when administered below the lesion (Fig. 8B). This illustrates the resilience of spinal circuitry, which remains functional below the lesion even in the cases of chronic and motor complete SCI. Conversely, participants classified as AIS A (i.e., P03 and P08) did not exhibit motor responses in hand muscles when TSS was applied below the lesion, indicating a disruption in spinal circuitry function. This lack of response can also be attributed to the prolonged duration of the injury, which might have led to the degeneration of motor pools located either at or below the level of SCI.

### Clinical and research implications

Depending on the severity of cervical SCI, affected individuals show a high variability in motor recovery following acute injury. The clinical and functional assessment tools (ISNCSCI and GRASSP) can be utilized as evaluating systems that (1) describe the level and the extent of injury based on a systematic motor and sensory examination of neurologic function and/or (2) outline residual motor and sensory function preserved following cervical SCI. Both ISNCSCI and GRASSP employ ordinal scales, which may be limited in sensitivity to subtle changes in recovery of function post SCI. Floor and ceiling effects, therefore, may present a challenge when using these assessment tools, as they are somewhat restricted in providing the complete picture of both impairment and recovery in sensory and motor pathways post cervical SCI. In turn, this can affect the extent to which these measures can be used to predict functional recovery as well as to develop rehabilitation programs post SCI.

Given the challenges with using solely a clinical approach in measuring sensory and motor function post cervical SCI, a comprehensive and detailed approach is needed to provide a complete picture of potential recovery of function post SCI, particularly as it relates to differences in individual’s UL function. The motor recruitment in UL muscles obtained from the assessment of evoked potentials above, at the site, and below the lesion using TSS contributes to the comprehensive understanding of the viability and function of selected UL motor pools. Utilizing the functional capabilities of neural networks above and below the level of paralysis is the key to restoring sensorimotor function for individuals with paralysis^16^. A novel treatment that combines multi-site electrical stimulation of the cervical spinal cord with targeted motor training can reactivate a part of the sublesional elements of the propriospinal network, thereby restoring UL motor function after paralyzing SCI^39^. However, in order to use this approach most effectively and in a targeted and individualized manner, it is vital to obtain comprehensive clinical, functional, and electrophysiological data to more fully understand the impairment level for an individual post cervical SCI. Once this comprehensive assessment occurs, future goals can shift to therapeutic intervention studies that will develop and maximize efficacy and utility of the integrated neurorehabilitation approach. In turn, this will support future clinical trials aimed at reversing neuromotor disorders, increasing independence and quality of daily living, and decreasing the cost of paralysis for individuals with these conditions.

### Limitations of study

This study’s limitations include a relatively small sample size that may compromise statistical power and subsequent interpretation of the results. Some variations in threshold and magnitude of the evoked potentials among different individuals can result from differences in the underlying skin resistance, amounts of subcutaneous fat, size of muscles, vertebral bone, intervertebral ligaments, and from the implanted spinal fusion hardware. Scar tissue at the site of stimulation can also divert the current, potentially contributing to uneven and asymmetrical activation of motor pools. Although we attempted to place the electrodes as symmetrically as possible, the placement of EMG electrodes may influence the bilateral recruitment of UL motor pools.

## Conclusion

Although clinical and functional assessments of residual sensorimotor pathways can be used to characterize the extent of residual motor function after SCI, electrophysiological assessments utilized in this study can provide a more comprehensive information of the functional state of spinal pathways after injury. The evaluation of evoked potentials above and below the lesion using TSS provides a substantial understanding of spinal circuitry and significantly enhances the ability to detect functional changes. These techniques are essential for ensuring the success of future clinical trials in rehabilitation training for individuals with SCI.

## Methods

### Participants

Eight participants with SCI (mean age, 32.8 $\pm$ 15.7 years; 1 female and 7 male) were enrolled in the study (Table 1). All participants gave informed consent to experimental procedures that were approved by the local ethics committee at Houston Methodist Research Institute in accordance with guidelines established in the Declaration of Helsinki. Participants with SCI had a chronic injury ($\ge$ 1 year) and were classified using the ISNCSCI examination as having a neurological level of injury (NLI) at or between C4 and C7 and American Spinal Cord Injury Association Impairment Scale (AIS) as AIS A (n = 3), AIS B (n = 3) and AIS C (n = 2).

**Table 1:** Demographics of spinal cord injury participants.

ID	Age (years)	Gender	AIS	NLI	Post-injury (years)	Hand dominance post injury
P01	67	Female	C	C4	18	Left
P02	50	Male	C	C6	4	Left
P03	25	Male	A	C4	14	Right
P04	29	Male	B	C4	7	Left
P05	27	Male	B	C6	2	Left
P06	23	Male	A	C4	4	Left
P07	19	Male	B	C5	4	Left
P08	22	Male	A	C4	4	Right

*AIS* American Spinal Cord Injury Association Impairment Scale, *NLI* neurologic level of injury.

### Clinical and functional assessment

To assess overall disability, we used the ISNCSCI NLI and AIS (ISNCSCI measurement; Table 2), which is the gold standard to determine the levels of injury and to classify the severity of the injury. UL motor function was comprehensively measured using subtests of the Graded and Redefined Assessment of Strength, Sensibility and Prehension^40^ (GRASSP v.1; Table 2). The GRASSP was designed as a clinical research tool to assess the degree of UL impairment in tetraplegia^41–43^. The right and left UL are tested separately. The subtests and items within subtests can be evaluated separately or as summed scores.

**Table 2:** Key upper-limb muscle groups (ISNCSCI) and upper-limb muscles (GRASSP assessment).

Segment	Spinal level	Key muscle groups ISNCSCI-ULMS	Muscles GRASSP-strength subtest
Upper arm Forearm	C5		Anterior deltoid
		Elbow flexors	Biceps brachii
	C6	Wrist extensors	Extensor carpi radialis
	C7	Elbow extensors	Triceps brachii
Hand			Extensor digitorum
	C8	Finger flexors	Flexor digitorum profundus
			Opponens pollicis
			Flexor pollicis longus
	T1	Finger abductors (little finger)	Abductor digiti minimi
			First dorsal interosseous

*ISNCSCI* International Standards for Neurological Classification of Spinal Cord Injury, *ULMS* upper-limb motor score, *GRASSP* graded and redefined assessment of strength, sensibility, and prehension, *C* cervical, *T* thoracic.

### Cervical transcutaneous spinal stimulation

During the experiments, the participants were in a seated position with their forearms supported anteriorly on a table. TSS was delivered using a constant-current stimulator DS8R (Digitimer Ltd, UK), via self-adhesive electrodes. Two cathode electrodes with a diameter of 3.2 cm (PALS, Axelgaard Manufacturing Co. Ltd., USA) were placed on the skin over the cervical spinal cord at midline between the spinous processes of C3–C4 (above lesion) and C7–T1 (below lesion) vertebrae. Two 7.5 × 13 cm self-adhesive oval anodes (PALS, Axelgaard Manufacturing Co. Ltd., USA) were placed on the anterior iliac crests of each participant.

Single-pulse TSS was delivered to record motor recruitment using a monophasic pulse of 500 µs duration. Stimulation began at 20 mA and was increased by increments of 5 mA to 150 mA or to the maximum tolerated intensity ensuring safety and the comfort of the participants.

### Transcranial magnetic stimulation

A Magstim BiStim 2002 magnetic stimulator (Magstim Co., Whitland, UK) connected to a double 70 mm figure-of-eight coil (Magstim, Co.) was utilized to deliver monophasic magnetic pulses over the primary motor cortex area projected to the UL muscles. The optimal position for eliciting the best motor response in UL muscles was established over the contralateral motor cortex with the coil held about 45 degrees to the mid-sagittal line (approximately perpendicular to the central sulcus). The position of the TMS coil was guided by Neuronavigation using Brainsight™ (Rogue Research, Montreal, QC, Canada). Guided by the Brainsight™ neuronavigation software, we calibrated the position of the coil and the position of the participant’s head in 3-D space using an infrared camera. Individual high-resolution structural MRI images were imported into the neuronavigation system. EMG data were collected bilaterally in UL muscles during each TMS trial to determine the motor hotspot and recruitment curves. TMS data for two participants (P01 and P02), both with AIS C, was not collected.

### Electromyographic recordings

Trigno Avanti wireless surface electromyography (EMG) electrodes (Delsys Inc., USA; common-mode rejection ratio < 80 dB; size: 27 × 37 × 13 mm^3^; input impedance: > 1015 Ω/0.2 pF) were placed bilaterally and symmetrically at six sites: biceps brachii (BIC), triceps brachii (TRI), flexor carpi radialis (FCR), extensor carpi radialis (ECR), first dorsal interosseous (FDI), and abductor pollicis brevis (APB) muscles. EMG data was amplified using a Trigno Avanti amplifier (Delsys Inc.; gain: 909; bandwidth: 20 to 450 Hz) and recorded at a sampling frequency of 2000 Hz using a PowerLab data acquisition system (ADInstruments, Australia).

### Magnetic resonance imaging

Structural magnetic resonance imaging (MRI) was used to confirm the location of SCI between C4 and C7 vertebrae in the participants. The images were acquired on a Siemens MAGNETOM Vida 3 T scanner (Siemens Healthineers, Germany) with a 64-channel head coil (Nova Medical Inc., USA). However, in the case of one participant (P05), a 20-channel coil was utilized due to their hair condition. T2-weighted turbo spin-echo sequence (TR/TE = 3000/104 ms, flip angle = 160°, FOV = 240 × 240 mm^2^, voxel size = 0.6 × 0.6 × 3.0 mm^3^), were acquired to evaluate spinal cord lesions. Sagittal sections were used to determine the extension of the lesions in reference to the vertebral bodies, and each segment was limited by the position of the intervertebral discs and confirmed by two observers. The caudal extension of the injury was marked along the extension of each vertebra taking as reference superior and inferior borders (Fig. 1).

### Data processing and analysis

Data were processed using LabChart Pro 8.1.13 software (ADInstruments, Australia). Recruitment curves were generated using the peak-to-peak EMG amplitude at each stimulation intensity for all tested muscles. The recruitment curves were obtained by increasing stimulation intensity by 5 mA starting at 20 mA to 150 mA or to the maximum tolerated intensity. For each muscle, recruitment values were subsequently normalized by the maximum value obtained for that specific muscle across all stimulation sites.

For each muscle, the response was calculated by measuring the peak-to-peak amplitude within a 20-ms window 5 ms from stimulus onset. Motor threshold (MT) was defined as the first amplitude that created a response greater than 20 µV^44^. To visualize the activation of UL motor pools during TSS delivered above and below the lesion, a heatmap was constructed by bilinear interpolation. A total of 55 color levels were used, with 5 major and 10 minor levels, using the MT stimulation intensity values during stimulation along the rostrocaudal axis. The maximum motor response (MaxR) was calculated as the average of the three largest evoked response values with the normalized motor recruitment values.

### Statistical analysis

All statistical analyses were performed using OriginPro 2023 (Origin Lab Corporation, Northampton, MA, USA). Assumptions of normality were tested using the Shapiro–Wilk test. Normally distributed data were analyzed using the two-way analysis of variance (ANOVA) to evaluate MT and MaxR differences between all tested UL muscles and TSS above and below the lesion. Data were corrected for multiple comparisons within figures using a Holm–Bonferroni’s *post-hoc* test. Non-normally distributed data were analyzed with a Kruskal–Wallis test followed by Dunn’s multiple comparison test. We conducted a Wilcoxon Signed-Rank test to analyze the ISNCSCI ULMS and GRASSP strength scores, comparing the left and right sides, as well as proximal and distal muscle groups. *p* values < 0.05 were considered significant (*p < 0.05, **p < 0.01 and ***p < 0.001).

Pearson’s correlation coefficient tests were conducted to investigate (1) the correlation between the two functional assessments: ISNCSCI ULMS and GRSSPS strength score, and (2) the relationship between the two functional assessments and TSS evoked motor responses for different cervical levels C5–C7 and C8–T1 distributions. The strengths of correlation coefficients were interpreted [direct (+) or inverse (−)] as weak (0.1–0.35), moderate (0.36–0.67), or strong (0.68–1)^45^.

### Supplementary Information

Supplementary Table S1.

## Data Availability

The datasets generated and analyzed during the current study are available from the corresponding author on reasonable request.
