# Supplementary material for: Characterizing neurological status in individuals with tetraplegia using transcutaneous spinal stimulation
Source: Sci Rep. 2023 Dec 6;13:21522. doi: 10.1038/s41598-023-48811-0 (PMC10700352; doi:10.1038/s41598-023-48811-0)
Supplement: Supplementary file 1 — Supplementary Table S1. [file 41598_2023_48811_MOESM1_ESM.docx]

**Table S1. Comprehensive ISNCSCI UL motor and GRASSP strength scores for individuals with spinal cord injury**

| **Spinal**  **Level** | **ISNCSCI**  **ULMS** | **GRASSP**  **Strength Subtest** | **P01** | | | | **P02** | | | | **P03** | | | | **P04** | | | | **P05** | | | | **P06** | | | | **P07** | | | | **P08** | | | | |
| --- | --- | --- | --- | --- | --- | --- | --- | --- | --- | --- | --- | --- | --- | --- | --- | --- | --- | --- | --- | --- | --- | --- | --- | --- | --- | --- | --- | --- | --- | --- | --- | --- | --- | --- | --- |
|  |  |  | **ISNCSCI** | | **GRASSP** | | **ISNCSCI** | | **GRASSP** | | **ISNCSCI** | | **GRASSP** | | **ISNCSCI** | | **GRASSP** | | **ISNCSCI** | | **GRASSP** | | **ISNCSCI** | | **GRASSP** | | **ISNCSCI** | | **GRASSP** | | **ISNCSCI** | | **GRASSP** | | |
|  |  |  | **L** | **R** | **L** | **R** | **L** | **R** | **L** | **R** | **L** | **R** | **L** | **R** | **L** | **R** | **L** | **R** | **L** | **R** | **L** | **R** | **L** | **R** | **L** | **R** | **L** | **R** | **L** | **R** | **L** | **R** | **L** | **R** |  |
| **C5** |  | **DEL** | - | - | 4 | 4 | - | - | 4 | 4 | - | - | 4 | 3 | - | - | 2 | 2 | - | - | 5 | 5 | - | - | 2 | 2 | - | - | 2 | 2 | - | - | 2 | 2 |  |
|  | **Elbow Flex** | **BIC** | 5 | 5 | 5 | 5 | 5 | 5 | 3 | 4 | 5 | 5 | 4 | 4 | 5 | 4 | 4 | 3 | 5 | 5 | 5 | 5 | 5 | 5 | 5 | 5 | 5 | 4 | 5 | 4 | 4 | 4 | 4 | 4 |  |
| **C6** | **Wrist Ext** | **ECR** | 4 | 4 | 4 | 4 | 5 | 5 | 4 | 4 | 4 | 5 | 5 | 5 | 2 | 0 | 3 | 1 | 5 | 5 | 5 | 5 | 4 | 3 | 5 | 2 | 4 | 0 | 4 | 0 | 0 | 2 | 0 | 2 |  |
| **C7** | **Elbow Ext** | **TRIC** | 2 | 2 | 3 | 2 | 4 | 5 | 4 | 4 | 1 | 1 | 2 | 2 | 0 | 0 | 2 | 1 | 5 | 3 | 5 | 3 | 3 | 4 | 3 | 3 | 3 | 3 | 3 | 3 | 0 | 0 | 0 | 0 |  |
|  |  | **ED** | - | - | 1 | 0 | - | - | 1 | 1 | - | - | 0 | 1 | - | - | 1 | 1 | - | - | 1 | 0 | - | - | 0 | 0 | - | - | 0 | 0 | - | - | 0 | 0 |  |
| **C8** | **Finger Flex** | **FDP** | 1 | 0 | 1 | 0 | 1 | 0 | 1 | 1 | 0 | 0 | 0 | 0 | 0 | 0 | 1 | 0 | 1 | 0 | 1 | 0 | 0 | 0 | 0 | 0 | 0 | 0 | 0 | 0 | 0 | 0 | 0 | 0 |  |
|  |  | **OP** | - | - | 1 | 0 | - | - | 1 | 0 | - | - | 0 | 0 | - | - | 1 | 0 | - | - | 0 | 0 | - | - | 0 | 0 | - | - | 0 | 0 | - | - | 0 | 0 |  |
|  |  | **FPL** | - | - | 1 | 0 | - | - | 1 | 1 | - | - | 0 | 0 | - | - | 2 | 1 | - | - | 1 | 0 | - | - | 0 | 0 | - | - | 0 | 0 | - | - | 0 | 0 |  |
| **T1** | **Finger Abd** | **ADM** | 1 | 0 | 1 | 0 | 1 | 0 | 0 | 1 | 0 | 0 | 0 | 0 | 0 | 0 | 1 | 0 | 0 | 0 | 0 | 0 | 0 | 0 | 0 | 0 | 0 | 0 | 0 | 0 | 0 | 0 | 0 | 0 |  |
|  |  | **FDI** | - | - | 0 | 0 | - | - | 1 | 1 | - | - | 0 | 1 | - | - | 1 | 0 | - | - | 0 | 0 | - | - | 0 | 0 | - | - | 0 | 0 | - | - | 0 | 0 |  |

ISNCSCI: International Standards for Neurological Classification of Spinal Cord Injury, 0 = total paralysis, 1 = palpable or visible contraction, 2 = active movement, full range of motion (ROM) with gravity eliminated, 3 = active movement, full ROM against gravity, 4 = active movement, full ROM against gravity and moderate resistance in a muscle specific position, 5 = (normal) active movement, full ROM against gravity and full resistance in a muscle specific position expected from an otherwise unimpaired person. Elbow Flex: elbow flexion, Wrist Ext: wrist extension, Elbow Ext: elbow extension, Finger Flex: long finger flexion, Finger Abd: small finger abduction.

GRASSP: Graded Redefined Assessment of Strength, Sensibility, and Prehension, version 1, Strength Subtest. 0 = No palpable or visible muscle contraction, 1 = Palpable or visible muscle contraction, 2 = Moves full ROM with gravity eliminated, 3 = Moves full ROM against gravity without added resistance, 4 = Holds position of resistance against moderate resistance, 5 = Holds position of resistance against maximal resistance. DEL: anterior deltoid, BIC: biceps brachii, ECR: extensor carpi radialis, TRIC: triceps brachii, ED: extensor digitorum, FDP: flexor digitorum profundus, OP: opponens pollicis, FPL: flexor pollicis longus, ADM: abductor digiti minimi, FDI: first dorsal interosseous.
